# Biochanin A Mitigates Oxidative Stress and Inflammation in Diabetic Myocardial Infarction: Insights From a Streptozotocin and Isoproterenol Rat Model

**DOI:** 10.7759/cureus.81455

**Published:** 2025-03-30

**Authors:** Umesh B Mahajan, Sameer Goyal

**Affiliations:** 1 Pharmacology, R. C. Patel Institute of Pharmaceutical Education and Research, Shirpur, IND

**Keywords:** biochanin a, diabetes mellitus in elderly, isoproterenol, nuclear factor erythroid 2-related factor 2 (nrf2), st-elevation myocardial infarction (stemi), streptozotocin

## Abstract

Introduction: Diabetes mellitus (DM) significantly increases the risk and severity of myocardial infarction (MI), contributing to poor cardiovascular outcomes through oxidative stress, inflammation, and endothelial dysfunction.

Methods: This study evaluates the cardioprotective potential of biochanin A (BCA), a naturally occurring isoflavonoid with antioxidant and anti-inflammatory properties, in a streptozotocin (STZ) and isoproterenol (ISO)-induced model of diabetic myocardial infarction (DMI) in rats. Male Wistar rats were divided into five groups, including normal controls, STZ+ISO, and three BCA-treated groups (5, 10, and 20 mg/kg).

Results: BCA administration significantly improved electrocardiographic parameters by reducing ST height and QT interval prolongation (p < 0.05). It also reduced myocardial injury markers (lactate dehydrogenase (LDH), creatinine kinase on myocardial bundle (CK-MB), aspartate transaminase (AST), and cardiac troponin-T (cTn-T)) in a dose-dependent manner (p < 0.001). BCA normalized blood pressure, heart rate, and left ventricular function, improving maximum and minimum rate of ventricular contraction (+dP/dt, -dP/dt) (p < 0.01). Furthermore, BCA significantly decreased blood glucose levels and improved lipid profiles by lowering total cholesterol (TC), triglycerides (TG), and low-density lipoprotein (LDL) while increasing high-density lipoprotein (HDL) levels (p < 0.001). It suppressed inflammation by reducing tumor necrosis factor (TNF)-α, interleukin (IL)-6, and IL-1β levels (p < 0.01). Oxidative stress markers, including malondialdehyde (MDA), were reduced, while antioxidant markers such as glutathione (GSH), superoxide dismutase (SOD), and catalase were increased (p < 0.001). BCA enhanced nuclear factor erythroid 2-related factor 2 (Nrf2) expression, promoting antioxidant defense. Histopathological analysis confirmed reduced myocardial injury and improved cardiac architecture.

Conclusion: These findings highlight BCA’s potential as a therapeutic agent for managing DMI through its dual antioxidant and anti-inflammatory actions.

## Introduction

Diabetes mellitus (DM) is a chronic metabolic disorder characterized by hyperglycemia resulting from defects in insulin secretion, action, or both [1]. Among its myriad complications, cardiovascular disease (CVD) stands as a leading cause of morbidity and mortality. Myocardial infarction (MI) in the diabetic population exacerbates this burden, as the presence of diabetes amplifies the severity of infarction, hinders myocardial repair, and accelerates the progression of heart failure [2]. Diabetic myocardial infarction (DMI) has a multifactorial etiology, involving disturbances in glucose metabolism, insulin resistance, oxidative stress, inflammation, and impaired endothelial function [3]. Diabetes impairs myocardial healing by altering fibroblast function and reducing angiogenesis, leading to poor tissue repair. Despite advancements in pharmacological interventions, the management of DMI remains suboptimal, necessitating the exploration of novel therapeutic agents. Additionally, existing therapies have limitations, such as side effects and an inability to directly target oxidative stress.

Biochanin A (BCA), a naturally occurring isoflavonoid predominantly found in red clover and other legumes, has emerged as a promising candidate for mitigating various diabetic complications [4]. This polyphenolic compound has garnered attention due to its potent antioxidant, anti-inflammatory, and cardioprotective properties. Notably, BCA has been shown to possess a wide range of biological activities, including the attenuation of oxidative stress and the modulation of inflammatory pathways, both of which are central to the pathogenesis of DMI. Preliminary studies suggest that BCA can reduce myocardial injury in various animal models of MI, although its specific role in DMI remains underexplored.

The hypothesis underlying this study posits that BCA exerts protective effects on the diabetic heart through its dual actions of combating oxidative stress and inflammation, both of which are key contributors to myocardial injury in diabetes. Streptozotocin (STZ)-induced diabetes, often used in experimental diabetes research, leads to oxidative damage and inflammatory responses, which are further aggravated by the addition of isoproterenol (ISO), a potent adrenergic agonist that mimics the pathological conditions of MI. This combined model of STZ and ISO-induced DMI is a reliable and widely accepted approach to study the pathophysiology of DMI and evaluate potential therapeutic strategies. Emerging evidence suggests that BCA can modulate several signalling pathways involved in oxidative stress and inflammation. In vitro and in vivo studies have demonstrated its ability to inhibit the activation of nuclear factor-kappa B (NF-κB), a key regulator of inflammation, and its capacity to scavenge reactive oxygen species (ROS) [5,6]. These actions potentially attenuate myocardial cell apoptosis, fibrosis, and dysfunction, which are commonly observed in diabetic heart disease. Additionally, BCA has been reported to improve lipid profiles, enhance endothelial function, and promote angiogenesis, all of which contribute to better cardiovascular outcomes.

While prior studies have highlighted the beneficial effects of BCA in non-diabetic models of MI, its effects in the context of DMI remain insufficiently characterized. Therefore, this study aimed to evaluate the therapeutic potential of BCA in STZ and ISO-induced DMI in rats, focusing on its ability to modulate oxidative stress, inflammation, and myocardial damage. By elucidating the molecular mechanisms through which BCA confers cardioprotection, this research sought to contribute to the development of novel, natural-based interventions for the management of diabetic heart disease.

## Materials and methods

Chemicals

BCA (≥ 98%) and STZ (≥ 98%) were procured from Sigma Aldrich, USA. The other required chemicals were purchased of analytical and laboratory grade from local vendors.

Experimental animals and protocol

Male Wistar rats (weighing between 170-200 g) were housed under controlled conditions with a 12-hour light/dark cycle and humidity of 50-60%. The rats had continuous access to purified water and were provided with multi-nutritional pellet feed (Nutrimix Laboratory Animal Feed, India). To minimize stress and ensure humane handling, animals were acclimated to handling procedures before the experiment, provided with environmental enrichment, and monitored regularly for any signs of distress. The Institutional Animal Ethics Committee (IAEC) of R. C. Patel Institute of Pharmaceutical Education and Research, Shirpur, India accepted and approved the protocol (CPCSEA/IAEC/RCPIPER/2017/32).

STZ and ISO-Induced DMI in Rats

STZ was dissolved in 0.1 M citrate buffer (pH 4.5) to maintain stability and enhance absorption. It was injected intraperitoneally at 55 mg/kg single dose [7], whereas ISO was dissolved in normal saline solution and injected subcutaneously on the 28th and 29th day [8,9].

Diabetes induction was confirmed by measuring fasting blood glucose levels after 72 hours of STZ injection, with rats having glucose levels ≥ 250 mg/dL considered diabetic.

Experimental Protocol

A total of 50 male Wistar rats were used in this study. The rats were randomly divided into five groups, with 10 rats in each group. The specifics of each group are outlined below:

About 0.5% carboxymethyl cellulose (CMC) and saline solution was used as a vehicle for BCA [10,11], and citric acid buffer solution was used for STZ [12,13].

Group 1. Normal: The rats were administered 0.5% CMC (1 ml/kg/day, orally) via gavage for a duration of four weeks and four times per week and 0.1 M citrate buffer (pH 4.5) solution (vehicle of STZ) (1 ml/kg, i.p.).

Group 2. STZ+ISO: The rats were administered 0.5% CMC (1 ml/kg/day, orally) via gavage and STZ (55 mg/kg, single dose i.p.) and ISO (85 mg/kg on 28th and 29th day, subcutaneously).

Group 3. BCA (5 mg/kg, p.o.): Rats were administered STZ (55 mg/kg, single dose i.p.) with BCA (5 mg/kg, p.o.) for four weeks and ISO (85 mg/kg on 28th and 29th day, subcutaneously). The dose of BCA was decided based on the previous studies. 

Group 4. BCA (10 mg/kg, p.o.): Rats were administered STZ (55 mg/kg, single dose i.p.) with BCA (10 mg/kg, p.o.) for four weeks and ISO (85 mg/kg on 28th and 29th day, subcutaneously).

Group 5. BCA (20 mg/kg, p.o.): Rats were administered STZ (55 mg/kg, single dose i.p.) with BCA (20 mg/kg, p.o.) for four weeks and ISO (85 mg/kg on 28th and 29th day, subcutaneously).

Electrocardiogram (ECG) and Hemodynamic Parameters

At the conclusion, the rats were anesthetized with urethane (1.25 g/kg, intraperitoneally). Each animal was placed in a supine position on an ECG board, and ECG recordings were obtained using a three-lead limb electrode configuration in accordance with standard preclinical protocols. The ECG recordings were continuously obtained using a data acquisition system (AD Instruments, Australia) [14]. 

Blood Pressure

The right carotid artery was cannulated, and the blood pressure was measured. Left ventricular end-diastolic pressure (LVEDP) and maximum and minimum rate of ventricular contraction (+dp/dt, -dp/dt) were recorded using PowerLab (AD Instruments, Australia). 

Estimation of Blood Glucose and Lipid Profile

Blood glucose and lipid profile were measured by using biochemistry methods as per the instructions given by manufacturers.

Estimation of Cardiac Injury Markers

Serum and plasma were stored at -20°C for subsequent measurement of cardiac injury markers. Levels of lactate dehydrogenase (LDH), creatinine kinase on myocardial bundle (CK-MB), aspartate transaminase (AST), and cardiac troponin-T (cTn-T) were determined according to the manufacturer's instructions (ERBA Diagnostics, India) [14].

Estimation of Oxidative Stress

The total protein content in the heart tissue homogenate was assessed using the Bradford protein assay as required for further calculations of oxidative stress parameters. Lipid peroxidation was evaluated by measuring the concentration of malondialdehyde (MDA) (thiobarbituric acid reactive substances (TBARS) assay). The level of reduced glutathione (GSH) in the heart tissue was determined. Catalase activity in the heart tissue was quantified, and superoxide dismutase (SOD) activity was also measured [15-17].

*Estimation of Nuclear Factor Erythroid 2-Related Factor 2* (*Nrf2)* *and Pro-inflammatory Cytokine Release*

Nrf2, along with the release of tumor necrosis factor (TNF)-α and interleukin (IL)-6, was measured using sandwich enzyme-linked immunosorbent assay (ELISA) kits, following the manufacturer's instructions (Abbkine, USA).

Histopathological Examination

The heart tissue was preserved in 10% neutral buffered formaldehyde. Specimens were then sectioned into 3-5 μm thick slices, embedded in paraffin, and stained with hematoxylin and eosin. The sections were examined under a light microscope (Motic, Canada) for signs of myocardial injury.

Statistical analysis

The data were presented as mean ± standard error of the mean (SEM) for each group. Statistical analysis was conducted using one-way analysis of variance (ANOVA), followed by Bonferroni’s post-hoc test. The data were analyzed using GraphPad Prism 8 software (GraphPad, California, USA).

## Results

Effect of BCA on electrocardiographic changes

**Figure 1:**
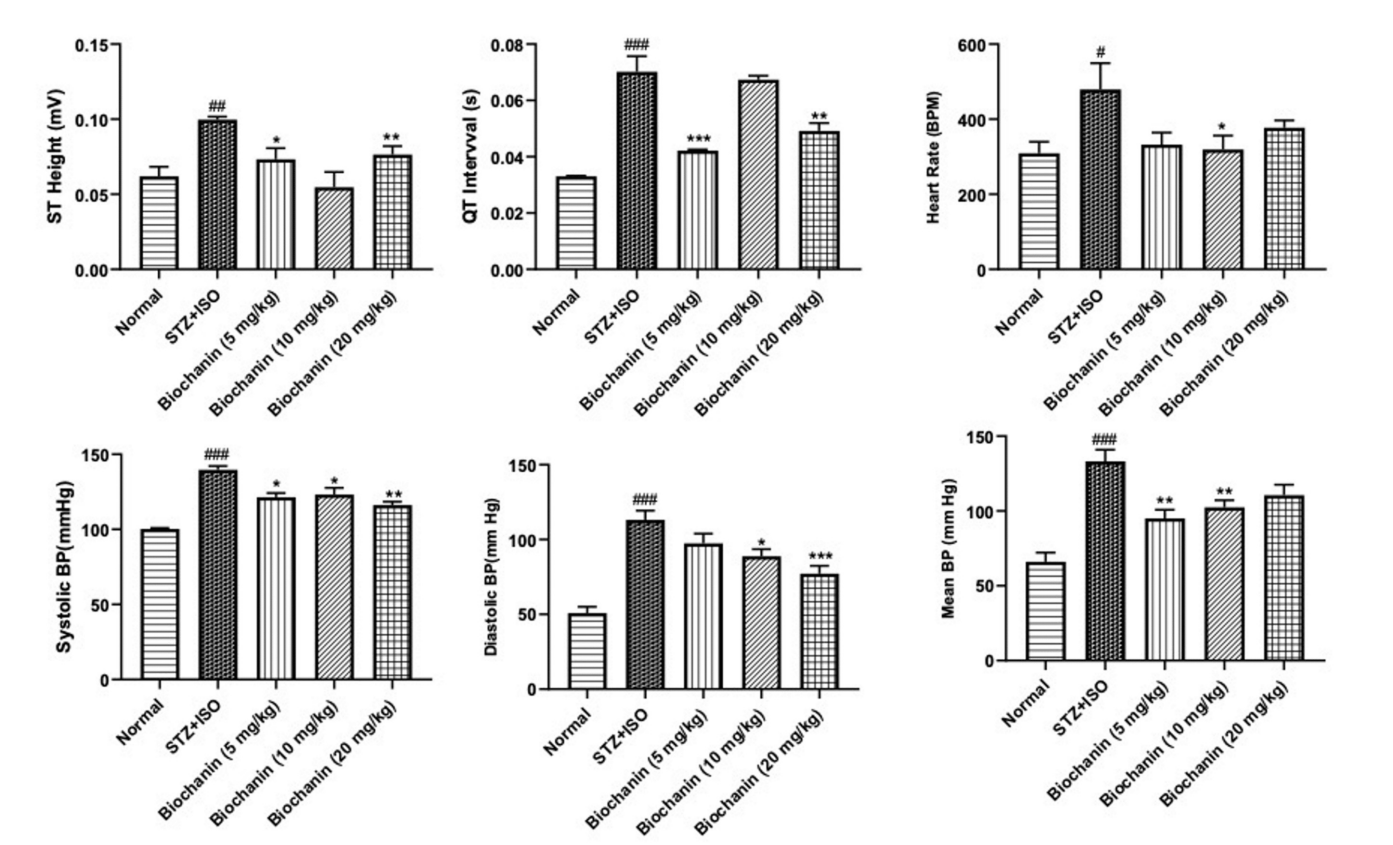
Effect of biochanin A on ECG and blood pressure in STZ+ISO-induced diabetes myocardial infarction in rats. The data are expressed as mean ± SEM (n = 6 per group). Statistical analysis was performed using one-way ANOVA, followed by Bonferroni’s multiple comparisons test. ST Height: ANOVA- P=0.2020, F=1.6, DFn, DFd 4, 25. QT Interval: ANOVA-P=0.0107, F=4.1, DFn, DFd 4, 25. Heart Rate: ANOVA-P=0.0227, F=3.4, DFn, DFd 4, 25. Systolic BP: ANOVA-P=0.2015, F=1.3, DFn, DFd 4, 25. Diastolic BP: ANOVA-P=0.9523, F=0.17, DFn, DFd 4, 25. Mean BP: ANOVA-P=0.8477, F=0.34, DFn, DFd 4, 25. ***p<0.001, **p<0.01, and *p<0.05 indicate significance compared to the STZ+ISO treated group. ##p<0.01 and ###p<0.001 denotes significance compared to the normal group. STZ: streptozotocin; ISO: isoproterenol; ANOVA: analysis of variance; BP: blood pressure; SEM: standard error of the mean; ECG: electrocardiogram

STZ+ISO-induced MI was associated with significant changes in ST height and QT interval. BCA reduced elevated ST height at all doses, but not to a significant extent. Whereas, prolongation of the QT interval was significantly reduced by BCA (p < 0.01) (Figure 1).

Effect of BCA on biochemical parameters

Administration of BCA significantly improved biochemical markers associated with DMI. The STZ+ISO group showed a marked increase in serum LDH, CK-MB, AST, and cTn-T levels, indicating severe myocardial injury and compromised cardiac function. BCA treatment at 5, 10, and 20 mg/kg resulted in a dose-dependent reduction in these markers. The STZ+ISO group exhibited significantly elevated LDH levels (58.48 ± 0.96 IU/L) compared to the normal group (25.22 ± 1.69 IU/L, p < 0.001). BCA administration reduced LDH levels to 28.72 ± 0.90 IU/L (5 mg/kg), 26.16 ± 1.29 IU/L (10 mg/kg), and 40.32 ± 1.50 IU/L (20 mg/kg). The same was observed in CK-MB levels, which increased significantly in the STZ+ISO group (226.8 ± 16.38 IU/L) versus the normal group (87.75 ± 8.19 IU/L, p < 0.001). BCA at the entire dose range restored CK-MB levels closer to normal (124.5 ± 10.87 IU/L and 120.1 ± 10.32 IU/L, respectively), indicating significant cardioprotection (p < 0.001). AST levels in the STZ+ISO group (47.14 ± 1.62 IU/L) were significantly higher than normal (18.38 ± 0.65 IU/L, p < 0.001). BCA significantly reduced AST levels at all doses. cTn-T levels were significantly elevated in the STZ+ISO group (29.58 ± 6.36 ng/L) compared to the normal group (5.54 ± 1.88 ng/L, p < 0.01). BCA reduced cTn-T levels to 11.34 ± 3.83 ng/L (20 mg/kg), suggesting improved myocardial integrity (p < 0.05), as shown in Table 1.

**Table 1:** Effect of biochanin A on biochemical parameters. Data are presented as mean ± SEM (n = 6/group). Statistical significance was determined using one-way ANOVA, followed by Bonferroni’s multiple comparisons test. LDH: ANOVA- P=0.1350, F=1.94, DFn, DFd 4, 25. CK-MB: ANOVA-P=0.7393, F=0.49, DFn, DFd 4, 25. AST: ANOVA-P=0.2186, F=1.55, DFn, DFd 4, 25. cTn-T ANOVA-P=0.2083, F=1.58, DFn, DFd 4, 25. ***p<0.001, **p<0.01, and *p<0.05 indicate significance compared to the STZ+ISO treated group. ##p<0.001 and ###p<0.001 denotes significance compared to the normal group. STZ: streptozotocin; ISO: isoproterenol; ANOVA: analysis of variance; LDH: lactate dehydrogenase; CK-MB: creatinine kinase on myocardial bundle; AST: aspartate transaminase; cTn-T: cardiac troponin-T; SEM: standard error of the mean

	LDH (IU/L)	CK-MB (IU/L)	AST (IU/L)	cTn-T (ng/L)
Normal	25.22 ± 1.69	87.75 ± 8.19	18.38 ± 0.65	5.54 ± 1.88
STZ+ISO	58.48 ± 0.96 ^###^	226.8 ± 16.38 ^###^	47.14 ± 1.62 ^###^	29.58 ± 6.36 ^##^
Biochanin (5 mg/kg)	28.72 ± 0.90 ^***^	124.5 ± 10.87 ^***^	40.57 ± 4.65	13.35 ± 3.27 ^*^
Biochanin (10 mg/kg)	26.16 ± 1.29 ^***^	152.1 ± 9.39 ^*^	34.42 ± 3.04 ^*^	10.81 ± 4.35 ^*^
Biochanin (20 mg/kg)	40.32 ± 1.50 ^***^	120.1 ± 10.32 ^***^	25.51 ± 2.20 ^***^	11.34 ± 3.83 ^*^

Effect of BCA on hemodynamic parameters

**Figure 2:**
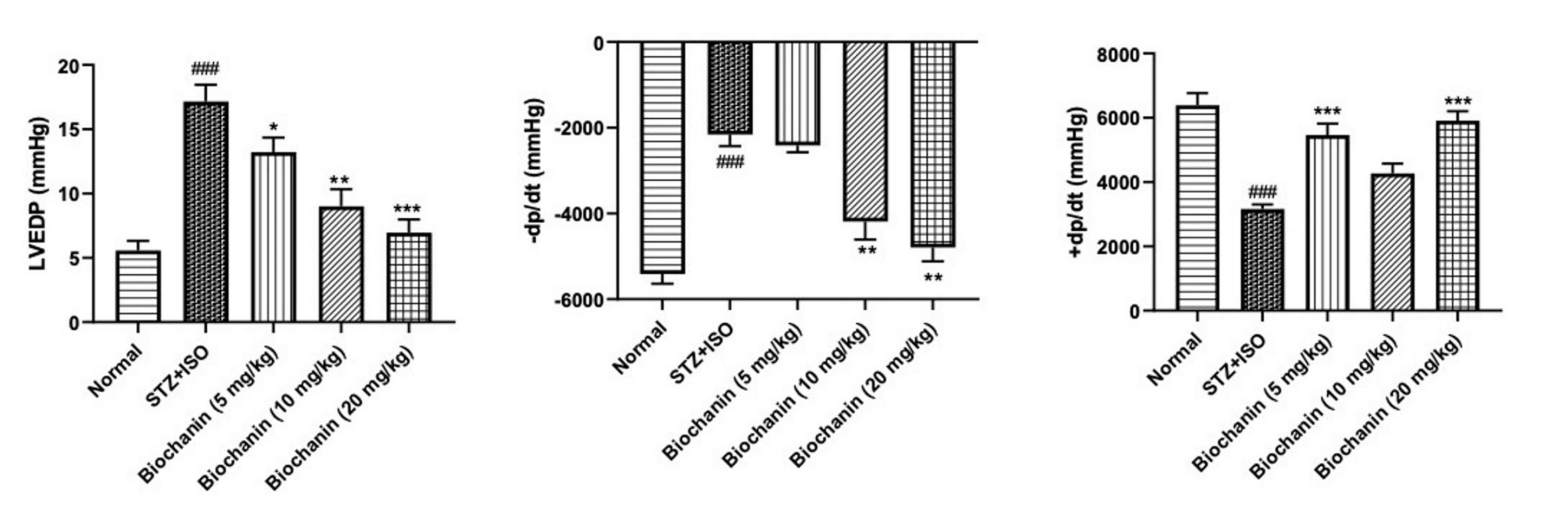
Effect of biochanin A on hemodynamic parameters and left ventricular pressure in STZ+ISO induced diabetes myocardial infarction in rats. Data are presented as the mean ± SEM (n = 6/group). Statistical significance was determined using one-way ANOVA, followed by Bonferroni’s multiple comparisons test. LVEDP: ANOVA- P=0.6905, F=0.56, DFn, DFd 4, 25. -dp/dt: ANOVA-P=0.3769, F=1.10, DFn, DFd 4, 25. +dp/dt: ANOVA-P=0.6091, F=0.68, DFn, DFd 4, 25. ***p<0.001, **p<0.01, and *p<0.05 indicate significance compared to the STZ+ISO treated group. ###p<0.001 denotes significance compared to the normal group. STZ: streptozotocin; ISO: isoproterenol; ANOVA: analysis of variance; SEM: standard error of the mean; LVEDP: left ventricular end-diastolic pressure; -dp/dt: minimum rate of ventricular contraction; +dp/dt: maximum rate of ventricular contraction

STZ+ISO significantly impaired cardiac function, increasing blood pressure and heart rate while reducing contractility. The systolic and diastolic blood pressure were reduced at all doses of BCA (p < 0.01). Elevated heart rate in the STZ+ISO group was normalized with BCA treatment (p < 0.05). LVEDP was significantly elevated in the STZ+ISO group, but BCA restored it in a dose-dependent manner (p < 0.05). +dP/dt and -dP/dt, the indicators of cardiac contractility, were improved significantly with BCA treatment (p < 0.01) (Figure 2).

Effect of BCA on blood glucose levels and lipid profile

STZ+ISO treatment caused significant alterations in the lipid profile and blood glucose levels, consistent with metabolic dysfunction. BCA treatment significantly restored these values toward normal levels. The STZ+ISO group showed severe hyperglycemia (278.2 ± 7.68 mg/dL) compared to the normal group (63.15 ± 4.26 mg/dL, p < 0.001). BCA significantly lowered blood glucose levels to 133.0 ± 8.80 mg/dL (5 mg/kg), 107.7 ± 6.86 mg/dL (10 mg/kg), and 207.3 ± 7.03 mg/dL (20 mg/kg). The high dose of BCA showed a lesser effect than 5 mg/kg and 10 mg/kg; this might be due to the feedback mechanism. Total cholesterol (TC) levels increased significantly in the STZ+ISO group (196.6 ± 7.15 mg/dL) compared to the normal group (114.8 ± 2.99 mg/dL, p < 0.001). BCA reduced TC levels at 5 mg/kg (157.2 ± 6.02 mg/dL) and 10 mg/kg (131.5 ± 7.93 mg/dL) (p < 0.001). Triglycerides (TG) levels increased significantly in the STZ+ISO group (170.4 ± 5.58 mg/dL) compared to normal (74.81 ± 2.09 mg/dL, p < 0.001). BCA lowered TG levels at 5 mg/kg (110.4 ± 7.51 mg/dL) and 10 mg/kg (83.01 ± 4.64 mg/dL) (p < 0.01). Also, BCA significantly reduced low-density lipoprotein (LDL) levels and increased high-density lipoprotein (HDL) levels (Table 2).

**Table 2:** Effect of biochanin A on blood glucose and lipid profile Data are presented as the mean ± SEM (n = 6/group). Statistical significance was determined using one-way ANOVA, followed by Bonferroni’s multiple comparisons test. TC: ANOVA- P=0.7497, F=0.48, DFn, DFd 4, 25. TG: ANOVA-P=0.3048, F=1.27, DFn, DFd 4, 25. LDL: ANOVA-P=0.0001, F=9.86, DFn, DFd 4, 25. HDL: ANOVA-P=0.8563, F=0.33, DFn, DFd 4, 25. ***p<0.001, **p<0.01, and *p<0.05 indicate significance compared to the STZ+ISO treated group. ###p<0.001 denotes significance compared to the normal group. STZ: streptozotocin; ISO: isoproterenol; ANOVA: analysis of variance; SEM: standard error of the mean; TC: total cholesterol; TG: triglycerides; LDL: low-density lipoprotein; HDL: high-density lipoprotein

	Blood glucose (mg/dl)	TC (mg/dl)	TG (mg/dl)	LDL (mg/dl)	HDL (mg/dl)
Normal	63.15 ± 4.26	114.8 ± 2.99	74.81 ± 2.09	25.22 ± 1.69	76.41 ± 4.34
STZ+ISO	278.2 ± 7.68 ^###^	196.6 ± 7.15 ^###^	170.4 ± 5.58 ^###^	58.48 ± 0.96 ^###^	51.33 ± 2.92 ^###^
Biochanin (5 mg/kg)	133.0 ± 8.80 ^***^	157.2 ± 6.02 ^***^	110.4 ± 7.51 ^**^	28.72 ± 0.90 ^**^	73.59 ± 2.08 ^***^
Biochanin (10 mg/kg)	107.7 ± 6.86 ^***^	131.5 ± 7.93 ^***^	83.01 ± 4.64 ^**^	26.16 ± 1.29 ^***^	70.63 ± 3.65 ^**^
Biochanin (20 mg/kg)	207.3 ± 7.03 ^**^	114.0 ± 6.14 ^***^	143.9 ± 7.79 ^*^	50.32 ± 4.96	44.59 ± 4.86

Effect of BCA on inflammatory markers

**Figure 3:**
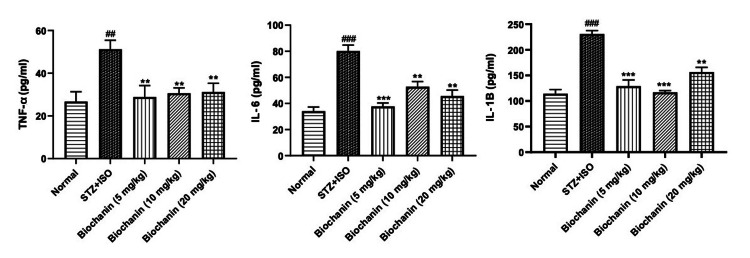
Effect of biochanin A on pro-inflammatory cytokines in STZ+ISO induced diabetes myocardial infarction in rats. Data are presented as the mean ± SEM (n = 6/group). Statistical significance was determined using one-way ANOVA, followed by Bonferroni’s multiple comparisons test. TNF-α: ANOVA- P=0.6346, F=0.66, DFn, DFd 4, 25. IL-6: ANOVA-P=0.5894, F=0.77, DFn, DFd 4, 25. IL-1β: ANOVA-P=0.0363, F=3.03, DFn, DFd 4, 25. ***p<0.001, **p<0.01, and *p<0.05 indicate significance compared to the STZ+ISO treated group. ###p<0.001 denotes significance compared to the normal group. STZ: streptozotocin; ISO: isoproterenol; ANOVA: analysis of variance; SEM: standard error of the mean; TNF: tumor necrosis factor; IL: interleukin

Inflammatory cytokines, including TNF-α, IL-6, and IL-1β, were elevated in the STZ+ISO group, indicating increased inflammation and myocardial injury. They increased from ~20 pg/mL (normal) to ~60 pg/mL (STZ+ISO). BCA treatment reduced TNF-α levels, with the 5 mg/kg and 10 mg/kg doses restoring them to normal. IL-6 and IL-1β showed a significant reduction with BCA at all doses, as shown in Figure 3.

Effect of BCA on oxidative stress

**Figure 4:**
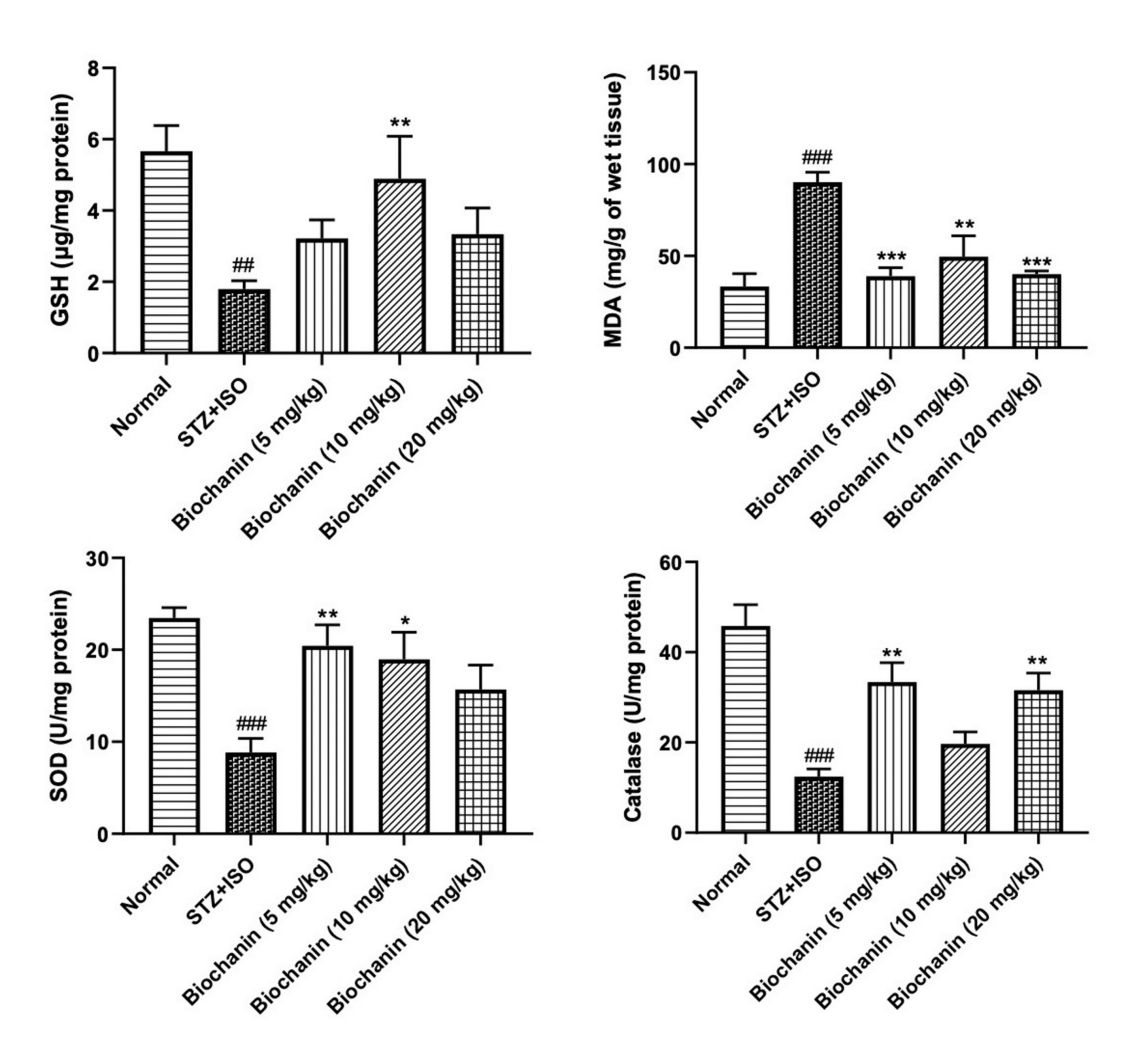
Effect of biochanin A on reactive oxygen species in STZ+ISO induced diabetes myocardial infarction in rats. Data are presented as the mean ± SEM (n = 6/group). Statistical significance was determined using one-way ANOVA, followed by Bonferroni’s multiple comparisons test. GSH: ANOVA- P=0.4840, F=0.89, DFn, DFd 4, 25. MDA: ANOVA-P=0.031, F=5.29, DFn, DFd 4, 25. SOD: ANOVA-P=0.5496, F=0.78, DFn, DFd 4, 25. Catalase: ANOVA-P=0.8393, F=0.35, DFn, DFd 4, 25. ***p<0.001, **p<0.01, and *p<0.05 indicate significance compared to the STZ+ISO treated group. ###p<0.001 denotes significance compared to the normal group. STZ: streptozotocin; ISO: isoproterenol; ANOVA: analysis of variance; SEM: standard error of the mean; GSH: glutathione; MDA: malondialdehyde; SOD: superoxide dismutase

Markers of oxidative stress, such as MDA, were increased in the STZ+ISO group, while antioxidant markers like reduced GSH, SOD, and catalase were reduced. MDA levels were elevated in the STZ+ISO group (~150 mg/g of wet tissue). BCA reduced MDA levels at all doses (p < 0.001). Post-hoc analysis showed that GSH, SOD, and catalase were increased with BCA treatment, suggesting improved redox balance and reduced oxidative stress (Figure 4).

Effect of BCA on Nrf2 levels

**Figure 5:**
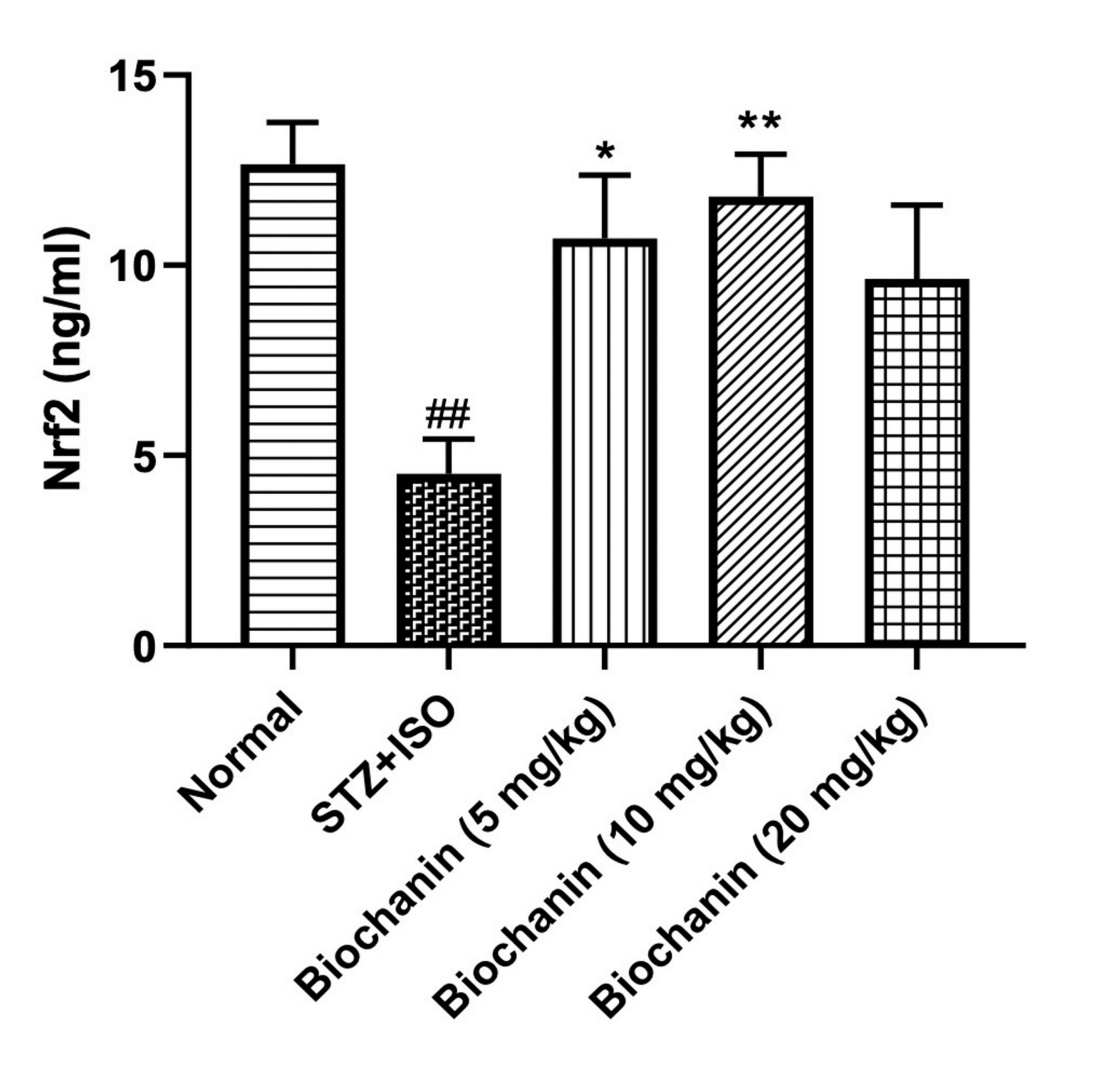
Effect of biochanin A on Nrf2 in STZ+ISO induced diabetes myocardial infarction in rats. Data are presented as the mean ± SEM (n = 6/group). Statistical significance was determined using one-way ANOVA, followed by Bonferroni’s multiple comparisons test. Nrf2: ANOVA- P=0.2825, F=1.34, DFn, DFd 4, 25. **p<0.01, and *p<0.05 indicate significance compared to the STZ+ISO treated group. ###p<0.001 denotes significance compared to the normal group. STZ: streptozotocin; ISO: isoproterenol; ANOVA: analysis of variance; SEM: standard error of the mean; Nrf2: nuclear factor erythroid 2-related factor 2

Nrf2 levels, which regulate the antioxidant defense pathway, were significantly reduced in the STZ+ISO group. BCA increased Nrf2 expression at all doses, indicating enhanced antioxidant response (p < 0.05). The highest dose of BCA increased the Nrf2, but not to a significant extent (Figure 5).

Effect of BCA on histopathology (hematoxylin and eosin staining)

**Figure 6:**
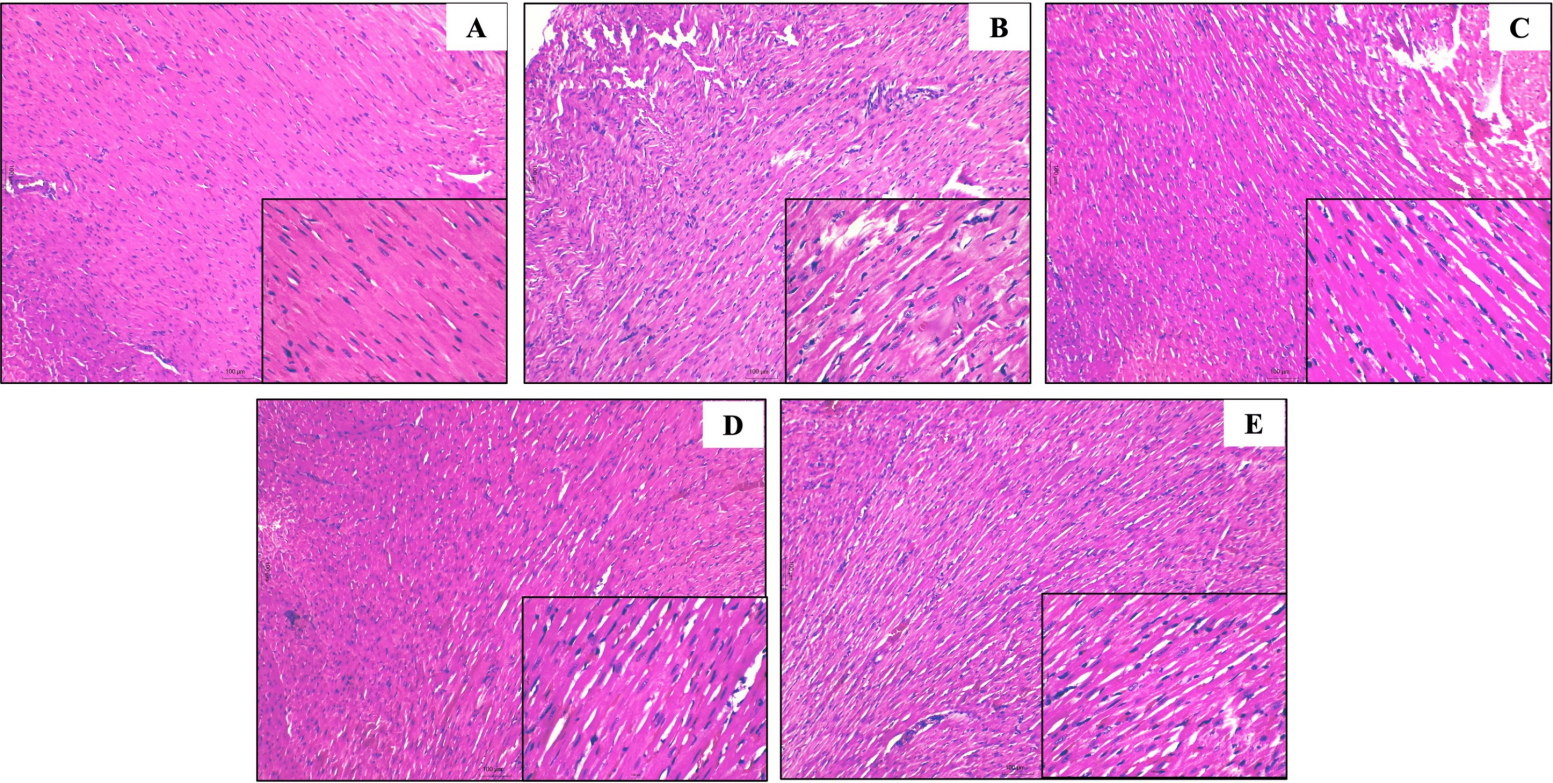
Effect of biochanin A on hematoxylin and eosin staining in heart tissues. A. Normal; B. STZ+ISO; C. Biochanin A (5 mg/kg); D. Biochanin A (10 mg/kg); E. Biochanin A (20 mg/kg). The images are shown in 100X and 400X (small square at the bottom right corner). STZ: streptozotocin; ISO: isoproterenol

Figure 6 presents histological images of cardiac tissue stained with hematoxylin and eosin from different experimental groups. The normal group showed well-organized, intact myocardial fibers with clear striations and minimal interstitial spaces, indicating normal cardiac tissue. The STZ+ISO group revealed significant myocardial damage, including disorganized muscle fibers, increased interstitial spaces, and infiltration of inflammatory cells, indicative of myocardial injury. In the BCA (5 mg/kg) group, partial restoration of myocardial structure was observed, with improved fiber alignment but some residual damage. The BCA (10 mg/kg) group exhibited more organized muscle fibers with reduced interstitial spaces and minimal inflammatory cell infiltration, suggesting a dose-dependent protective effect. The BCA (20 mg/kg) group showed nearly normal myocardial architecture with well-organized fibers and minimal damage, indicating a strong cardioprotective effect at this high dose (Figure 6).

## Discussion

The present study aimed to investigate the cardioprotective effects of BCA in a rat model of MI induced by STZ and ISO, with a focus on a range of biomarkers and physiological parameters related to cardiac function, oxidative stress, and metabolic disturbances. The results demonstrated that BCA treatment, particularly at the middle and high doses (10 and 20 mg/kg), provided significant improvements across multiple measured parameters. These findings suggest that BCA possesses potent therapeutic potential in managing diabetic-induced MI, likely mediated through modulation of oxidative stress and inflammation, along with the activation of the Nrf2 pathway.

The evaluation of key hemodynamic parameters - systolic blood pressure, diastolic blood pressure, and mean arterial pressure - revealed that BCA treatment effectively improved cardiac function, as evidenced by the normalization of these parameters in comparison to the STZ+ISO control group. Diabetes and MI often result in significant alterations in blood pressure regulation, contributing to further cardiac complications [18]. These results align with previous studies where natural compounds like flavonoids have demonstrated the ability to restore normal hemodynamics in the face of oxidative stress and myocardial injury [19,20].

Additionally, the ECG parameters showed significant improvements following BCA treatment, with reductions in arrhythmic events that are typically observed in DMI. The improvement in LVEDP further supports BCA’s beneficial effects on cardiac function, as LVEDP is an indicator of left ventricular compliance and heart failure progression [21,22]. Biochemical markers such as serum levels of LDH, CK-MB, AST, and cTn-T were significantly reduced in the BCA-treated rats, suggesting a decrease in myocardial injury and cellular damage. This observation corroborates the notion that BCA can mitigate the tissue damage induced by MI, possibly through its antioxidant and anti-inflammatory properties [23,24]. The improvements in the lipid profile, particularly with reductions in TC, TG, and LDL, and an increase in HDL, further indicate that BCA plays a role in modulating dyslipidemia, which is a common comorbidity in diabetic CVD. A dysregulated lipid metabolism in diabetes is known to exacerbate oxidative stress and inflammation, contributing to the progression of myocardial injury [25]. BCA’s effects on lipid metabolism could therefore be beneficial in reducing the cardiovascular risk associated with diabetes. Moreover, blood glucose levels were also significantly lower in the BCA-treated groups, which is consistent with previous reports on the antidiabetic effects of BCA [26]. This suggests that BCA not only improves cardiac outcomes but also has an indirect role in managing hyperglycemia, a well-established risk factor for myocardial infarction in diabetic patients.

One of the key mechanisms through which BCA exerts its cardioprotective effects appears to be the activation of the Nrf2 pathway. Nrf2 is a critical transcription factor that regulates the expression of antioxidant enzymes and plays a central role in cellular defense against oxidative stress [27]. Our findings of increased Nrf2 expression in the BCA-treated groups (in post-hoc analysis) indicate that BCA may counteract the heightened oxidative stress present in DMI by enhancing the cellular antioxidant capacity. This is in line with the previous literature that highlights the importance of Nrf2 activation in preventing and ameliorating CVDs, including MI [28]. By promoting Nrf2 signalling, BCA may mitigate the downstream effects of oxidative stress, including lipid peroxidation, protein damage, and inflammation, all of which contribute to the pathogenesis of MI in diabetic conditions [29]. Furthermore, the reduction in pro-inflammatory cytokines such as TNF-α observed in the BCA-treated rats further supports the involvement of anti-inflammatory mechanisms in the protective effects of BCA. TNF-α is a well-known mediator of inflammation that contributes to myocardial injury and dysfunction, and its reduction in response to BCA treatment suggests that BCA may attenuate the inflammatory cascade that exacerbates myocardial injury in diabetic rats [30]. The study highlights that the medium and highest doses (10 and 20 mg/kg) of BCA produced the most pronounced effects across all measured parameters, suggesting a dose-dependent relationship between BCA administration and its therapeutic efficacy except in some of the parameters like Nrf2, LDL, HDL, and TNF-α.

As a limitation, the present study lacks a long-term follow-up to assess the sustained effects of BCA treatment on cardiac function and overall health. Additionally, while the study provided promising results on the cardioprotective effects of BCA, the exact molecular mechanisms underlying these effects remain to be fully elucidated, and further studies are needed to clarify its precise interactions with other cellular pathways.

## Conclusions

In conclusion, the study presents compelling evidence that BCA provides significant cardioprotective effects in a rat model of DMI. The improvements in hemodynamic parameters, biochemical markers, lipid profile, and glucose regulation, along with the potential role of Nrf2 activation and the reduction of IL-6 and IL-1β, indicate that BCA could be a promising therapeutic candidate for managing diabetic cardiovascular complications. 
